# Corrigendum: The Rise of Populism and the Reconfiguration of the German Political Space

**DOI:** 10.3389/fdata.2021.833037

**Published:** 2022-01-11

**Authors:** Eckehard Olbrich, Sven Banisch

**Affiliations:** Max Planck Institute for Mathematics in the Sciences, Leipzig, Germany

**Keywords:** political space, network analysis, topic model (LDA), populism, party competition

In the original article, there was a mistake in the legends for the colors of the parties in Figures 2, 3 as published. The entries for the “Pirates” and the “Alternative for Germany” were interchanged. The figures with the corrected legends appear below.

**Figure 2:**
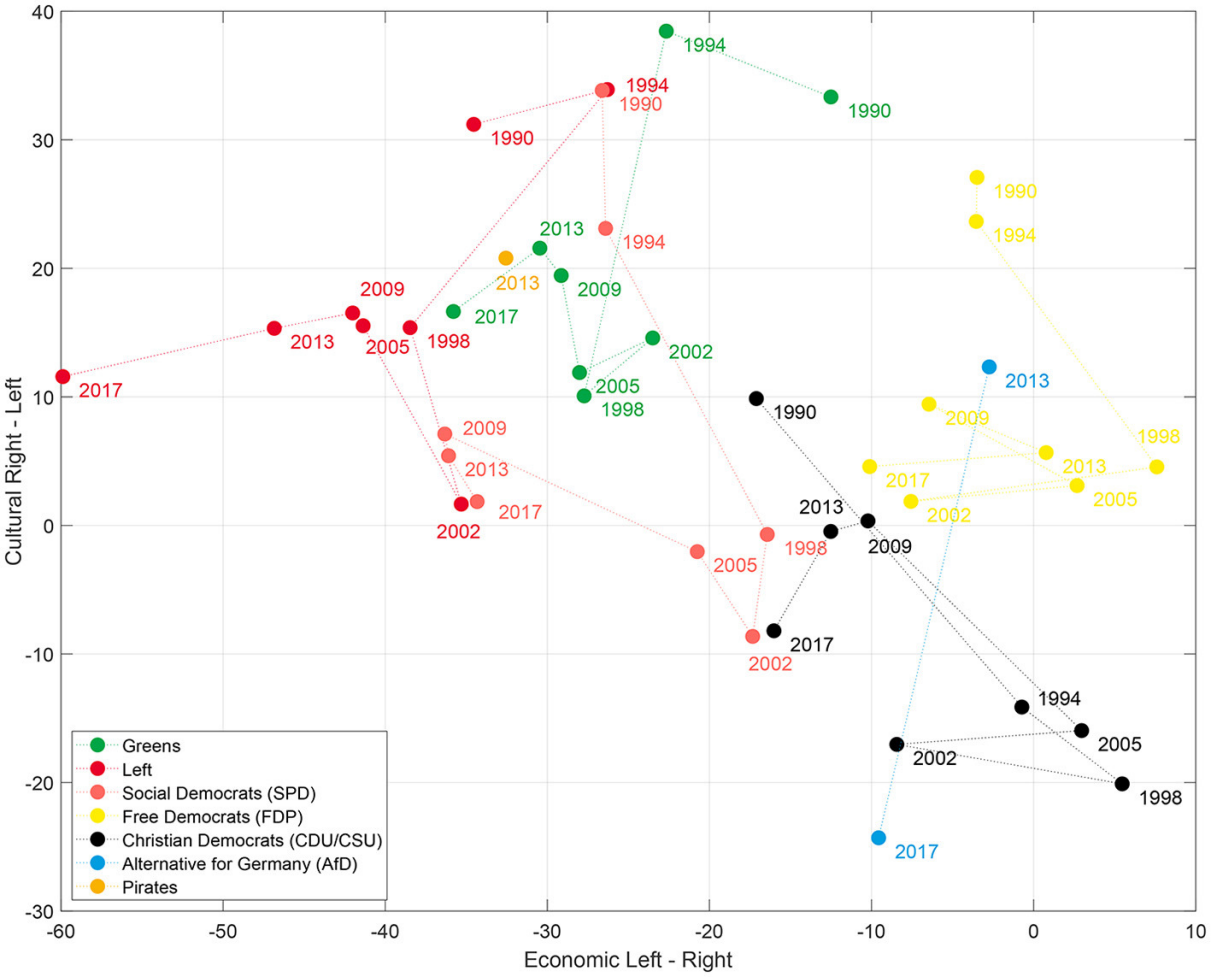
Projection on the 2-days political space with the economic and cultural axis spanned by the difference between the economic and cultural left and right categories, respectively (see Table 5) of the German parties between 1998 and 2017.

**Figure 3:**
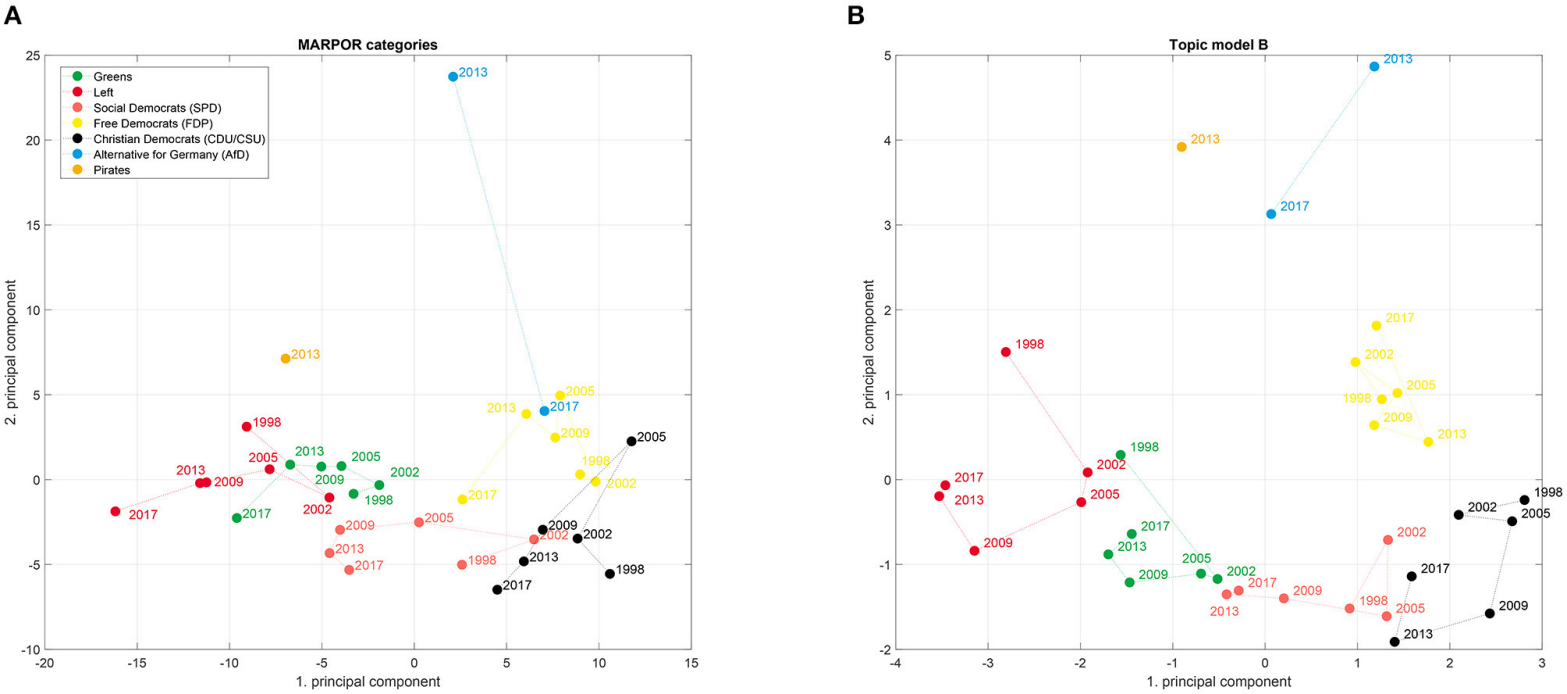
Political spaces spanned by the first and second PCA component from the MARPOR counts **(A)** and for the topic model B **(B)** for Germany 1998–2017.

The authors apologize for this error and state that this does not change the scientific conclusions of the article in any way. The original article has been updated.

## Publisher's Note

All claims expressed in this article are solely those of the authors and do not necessarily represent those of their affiliated organizations, or those of the publisher, the editors and the reviewers. Any product that may be evaluated in this article, or claim that may be made by its manufacturer, is not guaranteed or endorsed by the publisher.

